# The Health Equity and Effectiveness of Policy Options to Reduce Dietary Salt Intake in England: Policy Forecast

**DOI:** 10.1371/journal.pone.0127927

**Published:** 2015-07-01

**Authors:** Duncan O. S. Gillespie, Kirk Allen, Maria Guzman-Castillo, Piotr Bandosz, Patricia Moreira, Rory McGill, Elspeth Anwar, Ffion Lloyd-Williams, Helen Bromley, Peter J. Diggle, Simon Capewell, Martin O’Flaherty

**Affiliations:** 1 Department of Public Health and Policy, University of Liverpool, Liverpool, L69 3GB, United Kingdom; 2 Lancaster Medical School, Lancaster University, Lancaster, LA1 4YG, United Kingdom; UNC School of Dentistry, University of North Carolina-Chapel Hill, UNITED STATES

## Abstract

**Background:**

Public health action to reduce dietary salt intake has driven substantial reductions in coronary heart disease (CHD) over the past decade, but avoidable socio-economic differentials remain. We therefore forecast how further intervention to reduce dietary salt intake might affect the overall level and inequality of CHD mortality.

**Methods:**

We considered English adults, with socio-economic circumstances (SEC) stratified by quintiles of the Index of Multiple Deprivation. We used IMPACT_SEC_, a validated CHD policy model, to link policy implementation to salt intake, systolic blood pressure and CHD mortality. We forecast the effects of mandatory and voluntary product reformulation, nutrition labelling and social marketing (e.g., health promotion, education). To inform our forecasts, we elicited experts’ predictions on further policy implementation up to 2020. We then modelled the effects on CHD mortality up to 2025 and simultaneously assessed the socio-economic differentials of effect.

**Results:**

Mandatory reformulation might prevent or postpone 4,500 (2,900–6,100) CHD deaths in total, with the effect greater by 500 (300–700) deaths or 85% in the most deprived than in the most affluent. Further voluntary reformulation was predicted to be less effective and inequality-reducing, preventing or postponing 1,500 (200–5,000) CHD deaths in total, with the effect greater by 100 (−100–600) deaths or 49% in the most deprived than in the most affluent. Further social marketing and improvements to labelling might each prevent or postpone 400–500 CHD deaths, but minimally affect inequality.

**Conclusions:**

Mandatory engagement with industry to limit salt in processed-foods appears a promising and inequality-reducing option. For other policy options, our expert-driven forecast warns that future policy implementation might reach more deprived individuals less well, limiting inequality reduction. We therefore encourage planners to prioritise equity.

## Introduction

High blood pressure is the greatest contributor to the burden of coronary heart disease (CHD) worldwide [1] and high intakes of dietary salt (sodium) are a major contributor to high blood pressure [2–4]. For example, Mente et al. [4] analysed a prospective study of 18 high- and low-income countries: blood pressure increased with the amount of excreted sodium; the effect was stronger among older adults, those with hypertension, high body mass index, and high baseline levels of sodium excretion. Thus, reducing dietary salt intakes is at the forefront of global non-communicable disease strategies [5,6]. The World Health Organisation (WHO) have set the ambitious target for average intakes to fall to 5g/day by 2025, a substantial (~30%) reduction of current consumption in high-, middle-, and low-income countries [7]. This reduction should translate rapidly to substantial reductions in blood pressure and hence in the risk of death from stroke and CHD [8–10]. Population forecasts therefore predict substantial disease reductions from reducing dietary salt intake [11–13], including within the United Kingdom (UK) [14,15].

Previous policy strategies to reduce salt intakes at the population-level have targeted: (i) individual behaviour, through health promotion, information and education; (ii) the affordability and information displayed on processed foods; (iii) the amounts of salt added by industry to processed foods [5]. National strategies often include all the elements above, which we expect synergise to drive behaviour change [16]. Finland offers one exemplar for coordinated policy action. For over 30 years, the government and advisory bodies have improved individual awareness of the link between diet and health, nutrition labelling (to signpost healthy and unhealthy choices) and product regulation [17]. In the last decade, the UK has also pursued a coordinated approach, with the Change4Life awareness campaign, front-of-pack nutritional labelling, and the Responsibility Deal framework to support industry in producing healthier products [18–20].

Concurrently, salt intakes in the UK have fallen by an average 1.4 g/day since 2001, reaching 8.1 g/day in 2011 [20–22]. This has brought substantial disease reductions [23]. However, consumption still remains above the UK government’s target of 6 g/day and the WHO’s target of 5 g/day. Furthermore, significant socio-economic differentials in salt intakes remain (9% higher in manual worker compared with non-manual) [24]. Of concern is that socio-economic differentials in consumption do not appear to have narrowed over a decade of policy action [25]. For the United States, Bibbins-Domingo et al. [11] forecast that reductions in dietary salt intakes will also substantially reduce health inequalities. The public health community is therefore asking why inequality has not fallen, and how future action might be both effective and inequality-reducing.

New public health policies are therefore encouraged to more stringently assess how they might affect inequality [26]. This imperative to reduce socio-economic differentials was solidified by the 2010 Marmot review in the UK, which identified inequalities in diet as a key policy target [27]. Marmot emphasised that for success, the implementation of new interventions must have sufficient reach to all socio-economic groups.

However, in the design of interventions, doubt remains about how to reduce inequality most effectively. Recent theoretical analyses have distinguished interventions by the extent to which they are “structural”, by affecting our food environment, or “agentic”, by reliance on individual choice [28,29]. It appears that structural interventions, such as legislative and fiscal changes, tend to be both effective and inequality-reducing. However, a survey of policymakers in fourteen European countries indicated that most see achieving structural options as politically challenging [30]. Thus, agentic policy options are politically more likely, even though policymakers and academics agree that they have fewer benefits, and potentially widen inequality [31,32].

New assessments are therefore needed urgently to understand the potential overall effect and socio-economic differential of further action. We extend the validated IMPACT_SEC_ CHD policy model [33,34] to investigate how structural and agentic options might affect dietary salt intakes, and how inequality in effects might arise. As we will see, our model makes use of population survey data, published effect-sizes and expert-derived forecasts to project policy outcomes. We used it to forecast the potential impact on English adults of policies implemented during the 2015 UK parliament, projecting the health consequences to 2025.

## Methods

We extended the validated IMPACT_SEC_ CHD policy model for England [33,34]—a causal, deterministic, epidemiological model—to forecast up to 2025. Socio-economic circumstances (SEC) were measured by the English Index of Multiple Deprivation (IMD) [35]. The IMD summarises individual and community characteristics within Lower layer Super Output Areas (LSOAs) of approximately 1500 people. Based on the IMD, LSOAs were divided evenly among five quintiles; quintile one is the most affluent, and quintile five the most deprived.

We modelled a chain of links from further policy action to average daily salt intake, to systolic blood pressure (SBP), to the rate of death from CHD (codes 410–414 in the 9th, and I20–I25 in the 10th International Classification of Disease), stratified by IMD quintile, sex and age. Outcomes were the overall population effect, and the inequality in this effect, on the cumulative numbers of CHD deaths prevented or postponed, and the consequent life years gained up to 2025. We provide full model details in S1 Appendix.

### Policy modelling

We forecast how four policy options might further the reduction of salt intakes, over and above the progress on reducing dietary salt intakes already made by 2015:

1. The mandatory reformulation of processed-foods to reduce salt.
2. Further voluntary reformulation, which is dependent on the cooperation of specific companies.
3. Further individual-targeted social marketing, promotion and education around health and healthy eating.
4. Better labelling of product nutritional content, e.g., through front-of-pack traffic-light labels.

**Fig 1:**
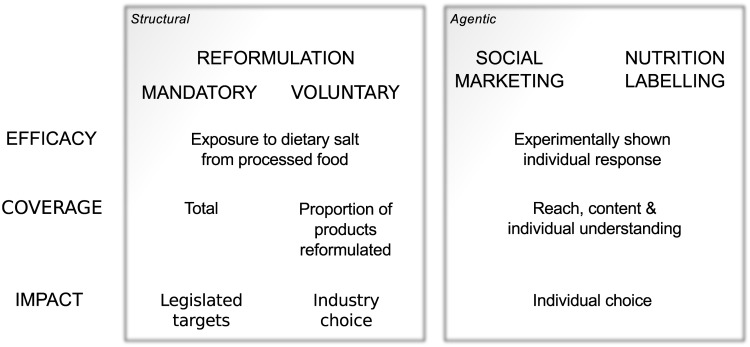
Steps to policy effects. We modelled the steps to the effect of an intervention on dietary salt intakes in terms of: Efficacy, the largest potential effect; Coverage, the spread of the intervention through the population; Impact, the size of the outcome that results, if the intervention reaches its target, considering industry or individual responsiveness. We developed this model based on the discussion surrounding McLaren et al. [29] who followed Giddens’ [37] description of society by distinguishing structural from agentic policy options. We further applied Tugwell et al.’s [36] concept that socio-economic differentials could arise at each step of policy action. In doing so, we expand the policy detail in Diderichsen et al.’s [38] description of the maintenance of inequality.

We modelled the policy effects on dietary salt intake in three steps, illustrated in Fig 1. We based our framework on the staircase analogy of Tugwell et al. [36]. In it, we define the steps:

1. **Efficacy**, the largest potential effect on dietary salt intake.
2. **Coverage**, the spread of the intervention through the population (the proportion of individuals reached by policy implementation).
3. **Impact**, the size of the outcome that results, if the intervention reaches its target (a proportion of the largest potential effect).

These steps combine to equal the effect on consumption. Importantly, socio-economic differentials can arise at each step and aggregate to the differential in the ultimate effect on consumption.

### Structural, reformulation options: Efficacy, Coverage and Impact

**Fig 2:**
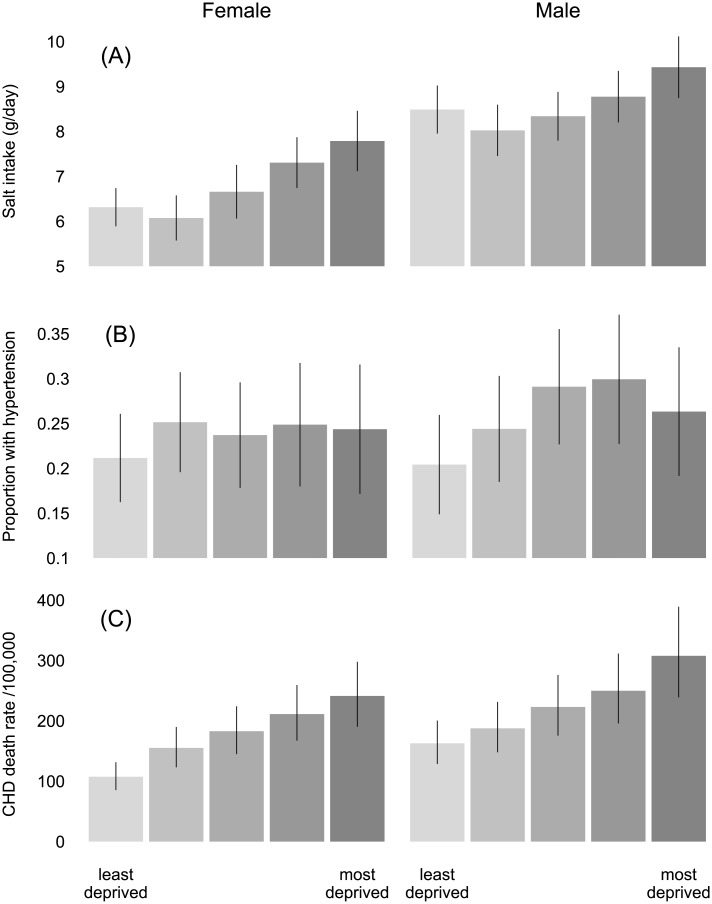
Socio-economic differentials. Baseline data for adults over 35 years, stratified by Index of Multiple Deprivation quintile: (A) dietary salt intake (g/day), converted from sodium per litre of urine recorded from the 2010 Health Survey for England; (B) the proportion of individuals with hypertension, i.e., blood pressure greater than or equal to 140/90 mmHg, from the 2011 Health Survey for England; (C) CHD death rates per 100,000 individuals (crude). Error bars show plus and minus one standard error around the mean estimates. See S1 Appendix.

For product reformulation, efficacy was the amount of salt (g/day) that individuals obtain from processed (including ultra-processed) foods. We estimated this value for each age, sex and IMD quintile in two-stages: (1) we estimated overall daily salt intakes from the 2010 Health Survey for England (HSE) (Fig 2A); (2) we then multiplied these intakes by the proportion derived from processed-foods, estimated from the 2011 Living Costs and Food Survey (LCFS). In this second stage, estimates were stratified by age only: 35–44 years, 0.66; 45–54 years, 0.68; 55–64 years, 0.69; 65–74 years, 0.69; 75+ years, 0.68. Compared to more general estimates [39], these values appear conservative.

Coverage and impact were respectively the proportion of products that saw some reformulation, and the consequent proportional salt reduction achieved. For mandatory reformulation, we designed a scenario where all products were reformulated (i.e., coverage = 1) and then investigated average reductions in salt content of 30% and 10% (i.e., impact = 0.3 or 0.1). Our forecast of voluntary reformulation was informed by experts’ forecasts of coverage and impact, as we outline below.

### Agentic, information-provision options: Efficacy, Coverage and Impact

For social marketing and nutrition labelling, efficacy was set at an average reduction in salt intakes of 2.39 g/day (95% Confidence Interval, 1.48 to 3.31 g/day). This is the effect after 3 to 36 months of receiving dietary advice, from a Cochrane systematic review and meta-analysis [40]. Whilst the effect of individualised advice is likely to be stronger than from population information campaigns, this is the best available data on an agentic intervention. We used it as the upper-limit to the effects on consumption.

The final effects of social marketing and nutrition labelling depended on their coverage and impact. Coverage was the proportion of individuals exposed to further social marketing and nutrition labelling; we defined an adult as exposed if they receive and understand a message sufficiently that they might persistently reduce consumption. We obtained forecasts of future coverage from our sample of experts. We defined impact as the proportion of the maximum possible reduction, i.e., as the proportion of the above value of efficacy, realised on average among the individuals reached. However, we had no data on this proportion, and so estimated our model at fixed values of 0.5 and 0.1.

### Socio-economic differentials in the effects of interventions

There is a paucity of studies on socio-economic differentials in effects of dietary interventions [41,42]. The reference set for different types of intervention is therefore small. This was highlighted recently by McGill et al. [41], in an update of the systematic review by Oldroyd et al. [42] of inequalities in the outcomes of healthy eating interventions. They found that the majority of studies screened did not explore socio-economic differentials. Agentic interventions (health promotion/information provision) appeared to have either a neutral or widening effect on inequalities. Structural interventions, which generally addressed the affordability and availability of healthy choices, tended to narrow inequalities. However, among the structural interventions only one addressed product reformulation. This study evaluated the UK’s policy action to reduce salt intake, which apparently has not had a detectable effect on socio-economic differentials [22,25].

We therefore also asked our sample of experts to anticipate the socio-economic differentials in future coverage and impact for voluntary reformulation, and in future coverage for social marketing and nutrition labelling.

### Forecasts of policy implementation by expert elicitation

We purposively selected a set of experts who shared a common involvement in research relating to the health-harms of dietary salt intake, and in the promotion of public health action to reduce dietary salt intakes (see S2 Appendix for further information on our expert elicitation). Each of our experts had published in the peer-reviewed academic literature on topics relevant to dietary salt intake and health, and many are active at the science–policy interface in this area. Their expertise was therefore mainly “substantive”, i.e., having an understanding of the general problem area, rather than “normative”, e.g., expertise in the processes of policy making, manufacturing or marketing.

Our initial sample contained 25 experts who (on 23^rd^ January 2014) were each emailed a brief introduction to our project together with an invitation to remotely complete a questionnaire survey. Those who agreed were then emailed the questionnaire (S3 Appendix), with a request to add annotations where they felt appropriate. Ethical approval was obtained from the Chair of the Ethics Committee of the Institute of Psychology, Health and Society, University of Liverpool (Reference: IPHS-1314-LB-267).

Our approach to expert elicitation focused on eliciting experts’ full range of uncertainty about future developments. We therefore asked each expert to provide “your best estimate, and also to suggest the absolute minimum value and maximum which you think could be true, include all possibilities except those that you consider extremely unlikely (less than 1% chance)”. Each expert was unaware of the other experts’ responses. We asked the experts for their judgements on seven questions, illustrated below.

For reformulation, we asked for future coverage with the question

*“What percentage of the processed food products currently consumed by an average English adult are likely to be reformulated to reduce salt by 2020*?*”*

and then for future impact by asking

*“In the processed foods which are reformulated to reduce salt by 2020*, *what percentage reduction is likely to be achieved*?*”*

For the agentic options, we first asked our experts to assume that coverage was currently 10%. The question we posed was

*“Assuming that 10% of the entire population of adults are currently sufficiently exposed to messages for persistent behaviour change*, *what is this percentage likely to be in 2020*?*”*

Then for socio-economic differentials, we elicited a linear differential across IMD quintiles by the question

*“If the value of your estimate was represented by 1*.*0 in the richest*, *what do you think would the value be in the poorest*?*”*

*(Putting `1*.*0’ would mean no different*, *`0*.*2’ would mean just 20% of that in the richest*.*)*

We received 13 completed questionnaires, with the last returned on 14^th^ March 2014. None declared a conflict of interest. We used PERT distributions [43] to linearly pool the experts’ judgements using probabilistic simulation (Table 1). Thus, as is recommended practice [44], all declared expert uncertainty propagated through to our model outputs.

**Table 1:** Expert forecasts of future policy implementation.

Policy	Component	Population-average	Socio-economic differential^a^
Voluntary reformulation	Coverage: Percentage of products reformulated	39% (9% to 82%)	0.79 (0.18 to 1.54)
	Impact: Percentage salt reduction	24% (9% to 46%)	
Social marketing	Coverage: New percentage exposure (considering 10% as the 2015 baseline)	22% (4% to 53%)	0.45 (0.15 to 0.90)
Nutrition labelling		23% (5% to 50%)	0.47 (0.08 to 1.12)

Means and 95% prediction intervals of the changes that our experts judged would characterise policy implementation by 2020. In S2 Appendix we present a detailed breakdown of the within and between expert variation in judgements about each parameter value.

^a^The answer to the question: “If the value in the most affluent is 1.00, what is the value in the most deprived? E.g., 0.90 would be a 10% decrease and 1.10 would be a 10% increase.”

### Linking salt intake to mortality

#### Baseline mortality projections

For each sex, age and IMD quintile, we forecast CHD mortality using a Bayesian estimation of an age, period and cohort model fitted to death rates from 1982 to 2006 [45]. We then multiplied these baseline rates by forecast population numbers from the Office for National Statistics (ONS).

#### Adjustment of baseline projections by policy effects

*Link 1*: *Salt intake to SBP*. We used the meta-analysis of He et al. [2], who conducted a Cochrane systematic review of randomised trials lasting at least four weeks. The relationship is a linear dose-response that is strongest in individuals with hypertension and weakens with age. Reducing salt intake by 6 g/day might cause SBP to fall by approximately 5.39 mmHg in adults with hypertension, and by some 2.42 mmHg in normotensive adults. Although He et al. did not investigate socio-economic variation, we weighted the effects by the prevalence of hypertension, which rises in more deprived areas (Fig 2B).

*Link 2*: *SBP to CHD death rates*. We used age- and sex-specific hazard ratios from the PSC meta-analysis of prospective studies [46]. Again, socio-economic variation was not investigated, but since hazard ratios describe a proportional relationship, a unit reduction in SBP will have a greater effect in more deprived groups, where CHD death rates are higher (Fig 2C).

*Time lags*. We incorporated two different time lags. First, we linearly phased-in the policy effects on salt intake up to 2020. Second, we modelled the physiological lag between decreased SBP and decreased CHD death rates. We assumed a lag of 4 years, which Kuulasmaa et al. [47] suggest separates changes in major risk factors and coronary events, based on data from 27 MONICA populations.

#### The mortality outcomes

Our outcomes were the number of deaths from CHD that might be prevented or postponed (DPPs), and the consequent life years gained. For life years gained, we first calculated remaining life expectancy by age, sex and IMD quintile using ONS data for 2012. We then multiplied this remaining life by the numbers of deaths prevented or postponed. We also computed premature DPPs, i.e., the number of CHD deaths averted up to age 75 years. For each metric, we report cumulative numbers from 2015 to 2025.

#### Quantifying socio-economic differentials in outcomes

We summarised the socio-economic differentials in the effect of each policy option among IMD quintiles using the Slope Index [48]. The Slope Index fits a linear gradient through the IMD-specific values and then reports the fitted value in the most affluent quintile minus that in the most deprived quintile. The result is therefore an indication of the absolute inequality in effects. For relative inequality, we divided the fitted value in the most deprived by that in the most affluent; values greater than one therefore indicate a greater effect in the most deprived. The advantage of the Slope Index is that it uses information from all socio-economic groups, yet straightforwardly reports inequality as a pairwise contrast between extreme groups.

### Uncertainty analysis

We analysed our model in the R environment (version 3.1.0) [49]. We used a Monte Carlo simulation that took 10,000 probabilistic samples from the distributions of expert-judgement and from the other parameters of our model for which we could quantify uncertainty. In all cases we used the median baseline forecast. We present means and 95% prediction intervals for the overall effects and the socio-economic differentials in effect. To show how experts’ judgements contributed to uncertainty, we re-estimated our model using only the mean of each expert-forecast parameter; we then calculated the percentage reduction in the 95% prediction intervals around the effects on CHD deaths.

## Results

### Upstream policy effects: dietary salt intakes

By 2020, we forecast that mandatory reformulation (with a high impact, 30% reduction in salt content) might reduce dietary salt intake by around 1.45 g/day (Table 2). Furthermore, the effect in the most deprived quintile would exceed that in the most affluent quintile by around 14% or 0.19 g/day, thus reducing inequality (Table 2). Our forecast also indicated that mandatory reformulation would be more inequality-reducing than further voluntary reformulation (Table 2). This is due to our experts’ anticipation that the future implementation of voluntary reformulation would reach more deprived individuals less well.

**Table 2:** Effects on salt intake and systolic blood pressure.

	Change to salt intake (g/day)			Change to systolic blood pressure (mmHg)		
	Population-average effect	Absolute differential in effect	Relative differential in effect	Population-average effect	Absolute differential of effect	Relative differential of effect
Mandatory reformulation (impact = 0.3)	-1.45 (-1.50 to -1.39)	-0.19 (-0.28 to -0.11)	1.14 (1.08 to 1.21)	-0.81 (-1.10 to -0.53)	-0.10 (-0.18 to -0.04)	1.14 (1.05 to 1.23)
Mandatory reformulation (impact = 0.1)	-0.48 (-0.50 to -0.46)	-0.065 (-0.095 to -0.035)	1.14 (1.08 to 1.21)	-0.27 (-0.37 to -0.18)	-0.035 (-0.060 to -0.012)	1.14 (1.05 to 1.23)
Voluntary reformulation	-0.48 (-1.58 to -0.08)	0.043 (-0.333 to 0.642)	0.90 (0.21 to 1.78)	-0.27 (-0.92 to -0.04)	0.024 (-0.185 to 0.350)	0.90 (0.22 to 1.76)
Social marketing (impact = 0.5)	-0.13 (-0.54 to 0.07)	0.093 (-0.079 to 0.518)	0.45 (0.15 to 0.90)	-0.078 (-0.326 to 0.043)	0.052 (-0.045 to 0.300)	0.46 (0.15 to 0.90)
Social marketing (impact = 0.1)	-0.027 (-0.108 to 0.014)	0.019 (-0.016 to 0.105)	0.45 (0.15 to 0.89)	-0.015 (-0.066 to 0.009)	0.011 (-0.009 to 0.062)	0.46 (0.15 to 0.90)
Nutrition labelling (impact = 0.5)	-0.16 (-0.51 to 0.06)	0.13 (-0.08 to 0.53)	0.46 (0.09 to 1.11)	-0.091 (-0.311 to 0.037)	0.071 (-0.048 to 0.308)	0.47 (0.09 to 1.12)
Nutrition labelling (impact = 0.1)	-0.031 (-0.100 to 0.013)	0.025 (-0.017 to 0.104)	0.46 (0.08 to 1.12)	-0.018 (-0.061 to 0.007)	0.014 (-0.009 to 0.061)	0.48 (0.10 to 1.13)

Mean effects assessed in year 2020, with 95% prediction intervals. For mandatory reformulation, impact is the proportional reduction in the salt content of processed foods. For social marketing and nutrition labelling, impact is the realised proportion of our specified maximum reduction in dietary salt intake. Absolute and relative socio-economic differentials of effect are based on the Slope Index. If the fitted value of the slope is *d* in the most deprived and *a* in the most affluent, then the absolute differential is *a*—*d* and the relative differential is *d* / *a*.

The agentic options, social marketing and nutrition labelling, might be a third as effective as voluntary reformulation, and a tenth as effective as our high impact mandatory scenario (Table 2). Despite uncertain effects, each option tended to widen inequality. This again comes from our experts’ anticipation that future implementation would reach more deprived individuals less well.

### Downstream policy effects: CHD mortality

Via the effects on SBP (Table 2), a high impact implementation of mandatory reformulation might prevent or postpone around 4,500 CHD deaths by 2025 (Table 3). There would consequently be a gain of around 44,000 life years (Table 3). In terms of premature deaths (before age 75 years), the same scenario might prevent or postpone around 1,400 CHD deaths by 2025 (Table 3).

**Table 3:** Mortality effects.

	Total CHD deaths prevented or postponed			Life years gained			Premature CHD deaths prevented or postponed		
	Total population effect	Absolute differential in effect	Relative differential in effect	Total population effect	Absolute differential in effect	Relative differential in effect	Total population effect	Absolute differential of effect	Relative differential of effect
Mandatory reformulation (impact = 0.3)	4,467 (2,854 to 6,147)	-528 (-748 to -325)	1.85 (1.61 to 2.14)	43,939 (29,388 to 58,750)	-8,143 (-11,031 to -5,311)	2.75 (2.31 to 3.28)	1,351 (909 to 1,810)	-339 (-458 to -226)	4.41 (3.58 to 5.44)
Mandatory reformulation (impact = 0.1)	1,502 (953 to 2,068)	-178 (-252 to -111)	1.85 (1.60 to 2.15)	14,791 (9,843 to 19,832)	-2,746 (-3,728 to -1,794)	2.75 (2.31 to 3.29)	455 (306 to 608)	-114 (-154 to -76)	4.42 (3.58 to 5.50)
Voluntary reformulation	1,474 (220 to 4,995)	-115 (-598 to 113)	1.49 (0.42 to 2.90)	14,372 (2,169 to 48,270)	-2,092 (-9,000 to 601)	2.19 (0.56 to 4.73)	438 (65 to 1,483)	-93 (-369 to 8)	3.51 (0.75 to 9.26)
Social marketing (impact = 0.5)	419 (-233 to 1,764)	10 (-100 to 165)	0.84 (0.34 to 1.61)	3,996 (-2,207 to 16,674)	-95 (-1,590 to 1,111)	1.08 (0.43 to 2.13)	123 (-68 to 516)	-9 (-71 to 22)	1.42 (0.53 to 2.92)
Social marketing (impact = 0.1)	84 (-47 to 355)	2 (-20 to 36)	0.85 (0.34 to 1.61)	780 (-442 to 3,362)	-18 (-319 to 237)	1.08 (0.43 to 2.13)	25 (-14 to 103)	-2 (-14 to 4)	1.42 (0.54 to 2.91)
Nutrition labelling (impact = 0.5)	489 (-194 to 1,697)	23 (-85 to 180)	0.87 (0.25 to 2.01)	4,641 (-1,791 to 15,985)	-1 (-1,423 to 1,188)	1.11 (0.31 to 2.69)	143 (-54 to 486)	-7 (-67 to 22)	1.48 (0.39 to 3.82)
Nutrition labelling (impact = 0.1)	98 (-38 to 334)	4 (-17 to 35)	0.88 (0.25 to 2.02)	928 (-355 to 3,121)	-2 (-291 to 235)	1.14 (0.32 to 2.71)	29 (-11 to 96)	-2 (-13 to 5)	1.51 (0.39 to 3.85)

Effects assessed cumulatively up to the year 2025, with 95% prediction intervals. For mandatory reformulation, impact is the proportional reduction in the salt content of processed foods. For social marketing and nutrition labelling, impact is the realised proportion of our specified maximum reduction in dietary salt intake. Absolute and relative socio-economic differentials of effect are based on the Slope Index. If the fitted value of the slope is *d* in the most deprived and *a* in the most affluent, then the absolute differential is *a*—*d* and the relative differential is *d* / *a*.

**Fig 3:**
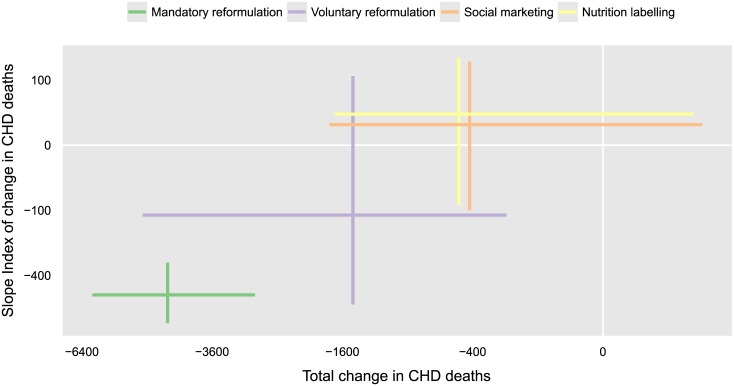
Policy effectiveness and inequality of effect. The cumulative changes to the total number of CHD deaths from 2015 up to 2025 (*x*-axis). We plot these against the socio-economic differentials in change (*y*-axis). Negative values for total change indicate fewer deaths. Negative values for the socio-economic differential indicate more deaths prevented or postponed in the most deprived, i.e., a reduction of inequality. Each axis is presented on a square-root transformed scale to better show the small effects of social marketing and nutrition labelling. Crosses indicate the 95% prediction intervals; where each set of vertical–horizontal lines cross, these are the mean predictions of effect.

Fig 3 contrasts mandatory reformulation to other policy options (see also Table 3). Further voluntary reformulation would have around a third less total effect than a high impact implementation of mandatory reformulation; it might prevent or postpone around 1,500 CHD deaths (Fig 3) and generate around 14,000 life years (Table 3). We forecast yet smaller effects for social marketing and nutrition labelling; each might prevent or postpone around 400–500 CHD deaths and generate around 4,000–5,000 life years.

#### Socio-economic differentials in mortality reduction

The *y*-axis in Fig 3 shows our forecast socio-economic differentials in the effects of each policy option. A high impact implementation of mandatory reformulation might prevent or postpone around 85% or 500 more CHD deaths in the most deprived than in the most affluent, reducing inequality (Table 3). Strikingly, inequality reductions were greater at young adult ages and were therefore more evident in terms of premature CHD deaths (Table 3). Despite more deprived quintiles having in general lower life expectancy, inequality reductions were also amplified in terms of life-years gained (Table 3).

Voluntary reformulation might prevent or postpone around 49% or 100 more CHD deaths in the most deprived than in the most affluent (Table 3), despite a minimal differential effect on salt intake (Table 2). This was due to the pre-existing socio-economic differentials in rates of hypertension and CHD death, which boosted the inequality-reduction. We forecast similar effects for social marketing and nutrition labelling, although these were highly uncertain (Tables 2 & 3).

### Uncertainty analysis

Our experts’ forecasts contributed upwards of 75% of the uncertainty in effects of further voluntary reformulation, social marketing and nutrition labelling. Thus, whilst we could not quantify all sources of uncertainty, the uncertainty in our forecast effects was dominated by uncertainty in our experts’ predictions of future policy implementation.

## Discussion

The mandatory reformulation of processed foods to reduce salt content has the potential to be both effective and inequality-reducing. Further voluntary reformulation, being optional on the part of industry, would still represent a substantial benefit but we forecast that it might only modestly narrow inequality in mortality. The agentic options, further social marketing and nutrition labelling, would likely bring substantially less benefit, and with highly uncertain effects on inequality in mortality.

### Strengths and limitations

#### Policy modelling

The main strength of this study was our incorporation of a theory-based model of policy action, which we combined with strong epidemiological evidence of health effects [3], and a thorough treatment of uncertainty. The steps in our policy model, “efficacy”, “coverage”, and “impact”, isolated different functional aspects of policy action. This was useful in isolating the components of policy implementation (which we then asked experts to forecast) from other characteristics of society that influence the ultimate policy effects on salt intakes. We were therefore able to gain an understanding of how inequality at each step aggregated to inequality in our outcomes.

However, there were also limitations. When modelling reformulation, we might have considered variation in the potential for reformulation among different types of processed food [50]. For many products substantial room for further salt reduction exists within the technical and safety requirements of manufacturing, e.g., for processed meats [51]. Indeed, between 2006 and 2011, changes in the salt content of UK products included decreases of 26% for convenience foods, and increases of 15% for processed vegetables, whilst processed meats showed little change [52]. There is also an agentic component to reformulation, which could modify outcomes, e.g., if individuals detect reformulation and consequently switch products or add discretionary salt. It may therefore be fruitful to link the effects of reformulation to individual consumption in more detail. However, since uncertainty remains as to the quantity of dietary salt that derives from different processed foods, and the demographic variation in this quantity, improvements to systems of data collection are also needed.

For the agentic policy options, we lacked data on individual responsiveness to the information they receive (our “impact” parameter). Whilst we investigated the influence of this responsiveness in sensitivity analysis, evidence on its magnitude and inequality would be valuable. It is concerning that an assessment of the UK’s Change4life campaign indicated that although awareness of the campaign increased, this translated only weakly to behaviour change [53]. We might also expect a high level of relative inequality in responsiveness [54]. Further studies of socio-economic differentials in individual responses to interventions should therefore be a priority, especially in light of the National Institute for Health and Care Excellence recommendation that new interventions be assessed for their potential inequality of effects [26].

It is also important to acknowledge that, in practice, each policy option will fit within a multi-component policy strategy, where policies undoubtedly interact. Our dietary salt consumption is, afterall, a product of our personal agency and the structure of our environment [29]. For example, “nudge” campaigns are likely to be enhanced by more supportive environments, e.g., by concurrent action on price, reformulation and labelling.

#### Use of expert forecasts

In our study, we obtained forecasts from experts because evidence on inequalities in the effect of dietary interventions was too sparse [41,42], and studies that do report inequalities in effect generally contain no information about the steps through which differential outcomes arise.

In designing our expert elicitation, we expected high uncertainty, as the range of possible future scenarios is vast and will not necessarily reflect the history of past policy outcomes. Our approach quantified the full range of declared expert uncertainty, without subsequent discussion among experts. This is in contrast to Delphi-based approaches, which aim to reach a more precise consensus [55]. Famously, an expert-driven forecast of climate change was criticised for projecting too certain a future, due to forecasting based on the experts’ consensus rather than presenting the full range of expert uncertainty [56]. The advantage of our approach is therefore that it faithfully represents expert uncertainty. Indeed, a recent review of health economics studies indicates that expert uncertainty is rarely quantified as robustly as we have done here [57].

However, the use of experts does mean that our forecast is sensitive to the characteristics of the experts selected. All our experts shared an academic interest in promoting public health through dietary salt reduction. Their judgements are therefore likely to differ from other pools of experts, e.g., those with more normative expertise of the policy or industrial processes. Further studies might therefore wish to explore the differences among expert groups, e.g., academics vs. industry. Collecting extensive qualitative data—e.g., from follow-up phone calls—would then help interpret emergent splits in opinion. This might prove to be informative, as industry has vested interests in future public health strategy [58], including in regard to dietary salt intake [59]. Within industry there is also specialist technical and economic expertise, including experts on consumer behaviour. When surveying industry experts, it may therefore be possible to ask more detailed questions, e.g., on the future variation in reformulation to reduce salt content among different categories of processed food.

#### Public health implications

The principle individuals to benefit from the reformulation of processed foods to reduce salt are the individuals who consume the most salt from processed foods. In the UK, salt consumption tends to rise among individuals living in areas of greater deprivation, with lower levels of education or occupational status [24]. Thus, reformulation approaches have a natural tendency to reduce inequalities in nutrition and health.

However, two factors might mean that reformulation does not produce the expected inequality reduction. First, either the structural or agentic aspects of society might shift over time to produce new socio-economic differentials. For example, in Canada new inequalities in table-salt use have arisen [60]. Second, as our experts’ forecast indicated, reformulation might reach certain parts of society less well. For example, if the products reformulated tend to be consumed by more health-conscious individuals, with already low salt intakes. In this case, mandatory reformulation is a clear pathway to increased equity.

Indeed, our forecast suggests that the persistence of socio-economic differentials in salt intake in the UK [25] is due, in part, to a strategy of voluntary rather than mandatory reformulation. Yet there is great potential for future public health action to reduce inequality, given that implementation has sufficient reach to individuals with the highest salt-intakes. As part of a coordinated policy strategy, featuring structural and agentic approaches, we emphasise that mandatory reformulation would be effective and inequality-reducing. It is also likely to be the most cost-effective option [61].

The epidemiological evidence that reductions in dietary salt intake would consequently reduce blood pressure and mortality is now increasingly consistent, despite methodological issues in some studies [3]. In addition to CHD, changes in salt intake are likely to affect other diseases, such as stroke [9] and chronic kidney disease [62]. Future work might therefore aim to model multiple disease outcomes, to more accurately represent the total and socio-economic differential of health benefits. Interestingly, modelling inequalities in multiple aspects of health can lead to counter-intuitive outcomes [63].

The aetiology of CHD also includes a wide array of nutrition and lifestyle factors. However, we have modelled only one aspect of nutrition (dietary salt intake), but other aspects, e.g., potassium from fruits and vegetables, likely modify the health effects of elevated salt intakes [4]. Our outcomes may therefore differ if interventions alter nutrition in complex ways. Furthermore, since nutritional variation among socio-economic groups is complex, covering salt, sugar, fruit and vegetables, meat products etc. [64], more detailed modelling of nutrition might also modify the anticipated effects on inequalities. There is therefore great potential for further research to support the coordinated development of public health policy over multiple aspects of nutrition.

## Conclusions

A policy emphasis on mandatory reformulation to reduce the salt in processed foods would likely be an effective and inequality-reducing route to improving population health. Policymakers and practitioners should, however, be invited routinely to consider the impact of their planned interventions on inequalities, perhaps using qualitative approaches [65]. If this is done, then our forecast indicates that future national strategies to reduce salt intake will become substantially more effective and equitable.

## Supporting Information

S1 Appendix Modelling methods. (PDF)

S2 Appendix Expert elicitation methods and detailed results. (PDF)

S3 Appendix Expert elicitation questionnaire. (PDF)
