# Correction: The Health Equity and Effectiveness of Policy Options to Reduce Dietary Salt Intake in England: Policy Forecast

**DOI:** 10.1371/journal.pone.0134064

**Published:** 2015-07-24

**Authors:** Duncan O. S. Gillespie, Kirk Allen, Maria Guzman-Castillo, Piotr Bandosz, Patricia Moreira, Rory McGill, Elspeth Anwar, Ffion Lloyd-Williams, Helen Bromley, Peter J. Diggle, Simon Capewell, Martin O’Flaherty

There is information missing from funding section of this paper. The funding section should read: This article presents independent research funded by the National Institute for Health Research School for Public Health Research (NIHR SPHR) through a grant (SPHR-LIL-PH1-MCD) to the LiLaC collaboration between the University of Liverpool and Lancaster University. The views expressed are those of the authors and not necessarily those of the NHS, the NIHR or the Department of Health.
