# Supplementary material for: The Health Equity and Effectiveness of Policy Options to Reduce Dietary Salt Intake in England: Policy Forecast
Source: PLoS One. 2015 Jul 1;10(7):e0127927. doi: 10.1371/journal.pone.0127927 (PMC4488881; doi:10.1371/journal.pone.0127927)
Supplement: S1 Appendix — (PDF) [file pone.0127927.s001.pdf]

Appendix 1 for "The health equity and effectiveness of policy options to reduce dietary salt intake in England: policy forecast"

Duncan Gillespie, Kirk Allen, Maria Guzman-Castillo, Piotr Bandosz, Patricia Moreira, Rory McGill, Elspeth Anwar, Ffion Lloyd-Williams, Helen Bromley, Peter Diggle, Simon Capewell, and Martin O'Flaherty

Contents

1 Health Survey for England data **1**

2 Living Costs and Food Survey data **2**

3 CHD death rates **2**

4 Population counts **2**

5 New addition: the policy layer **2**
5.1 Mathematical structure 3
5.1.1 Reformulation of processed foods 3
5.1.2 Social marketing 4
5.1.3 Nutrition labelling 4

6 IMPACT$_{\text{SEC}}$: the epidemiology layer **5**
6.1 Link 1: Salt intake to SBP 5
6.2 Link 2: SBP to CHD death rates 6

7 Outputs **6**
7.1 Aggregate outcomes and the socio-economic differential 6
7.2 Life-years gained 6
7.3 Uncertainty analysis 7
7.3.1 Assumptions of independence in draws of related parameters 7
7.3.2 Uncertainty decomposition 8

8 Data tables **8**

1 Health Survey for England data

We took two variables from the Health Survey for England (HSfE), each stratified by age, sex and quintiles of the Index of Multiple Deprivation (IMDQ): urinary sodium and the proportion of individuals with hypertension.

The HSfE is an annual nationwide household survey, representative of the English population [1]. Households were invited to participate if drawn at random from the Postcode Address File; annually, around 70% of households responded. Data was collected during a nurse visit to the households that agreed.

We accessed the HSfE data via the UK Data Service's Nesstar catalogue. For urinary sodium, we applied the urine analysis sample weights; for proportion hypertensive (downloaded directly as a derived

variable) we used the core nurse sample weights. We converted urinary sodium (mmol/litre) to salt, i.e., sodium chloride (grams/litre), using

$$100 \text{ mmol sodium} = 5.85 \text{ grams salt.} \tag{1}$$

We then scaled this value to give the salt consumed in 24 hours, as follows. As a reference, we used the value of 8.1 g/day salt from a 24 hour urine analysis of English adults aged 19–64 in 2011 [2]. Then, to make our value for mean consumption approximately equal this reference, we assumed that adults passed an average 1.5 litres of urine per day.

2 Living Costs and Food Survey data

We used the Living Costs and Food Survey (LCFS) 2011 to estimate age-specific proportions of average daily salt intake obtained from processed foods. We classified food purchases in the LCFS into processed and ultra-processed foods vs. other categories of food. We then used nutrition profiles of each food to derive an estimate of average daily salt intake.

However, compared to the reference value of 8.1 g/day [2], average salt intake appeared to be underestimated by approximately 2 g/day. We assumed that this additional 2 g/day derived from an unknown source, and added it to the denominator in our calculation of the proportion of salt from processed foods. Our estimates are therefore likely to be conservative.

3 CHD death rates

We used the same data on CHD death rates as Bajekal et al. [3]. As described in [3], death counts were matched via area of residence to the corresponding deprivation quintile by the Office for National Statistics (ONS), before being released for research purposes. These data were sex and IMDQ specific central death rates from CHD (codes 410–414 in the 9th version and I20–I25 in the 10th version of the International Classification of Diseases). Data were provided in 10×1 age and period form, for ages 35 to 85+ and years 1982 to 2006.

Using these data, we conducted a forecast, from which we took the rates up to 2025; full details on the forecast are presented in [4]. Briefly, to model and project CHD death rates, we used an age-period-cohort (APC) model, fitted separately to each sex and IMDQ. Analysis was conducted using the BAMP software [5], which uses a Bayesian approach to fit an APC model to death rates on the logit scale. We used the median forecast as our mortality baseline.

4 Population counts

We downloaded sex-specific forecast population numbers for England from the Office for National Statistics (file 1, file 2) on 24 February 2014; we used the scenario of constant fertility from the jump-off year of 2012. To stratify the population numbers by IMDQ, we assumed that for each age and sex, the population distribution across the five IMDQs remained as it was in 2007.

5 New addition: the policy layer

We identified four policy options that could further reduce dietary salt intake:

- ***Mandatory reformulation***. Enforced engagement with industry to reduce the salt content of processed foods.
- ***Voluntary reformulation***. Furthering the current strategy to encourage industry to voluntarily reformulate to reduce salt, under the Department of Health's Responsibility Deal.
- ***Social marketing***. The promotion of effective healthy eating and lifestyle messages, which might be general or salt-specific, e.g., through the Change4Life campaign.

- ***Nutrition labelling***. Improvement of the display and public understanding of nutritional information displayed on processed foods. This includes developments in rear-of-pack and front-of-pack labelling, e.g., through the traffic-light system.

In Appendix S2, we give a detailed description of our methods and results for the use of expert judgements to inform the forecast effects of these policy options.

5.1 Mathematical structure

We extended Tugwell et al.'s [6] "staircase" framework for how socio-economic differentials might enter at different steps in the pathway from an intervention's implementation to its eventual effect. At each step (in our case the steps were, *efficacy*, *coverage* and *impact*, see below) socio-economic differentials might arise and aggregate to a differential in the final outcome.

Here we present the mathematical formulae used.

Table 1: **Symbols used to describe the policy layer**. We indicate stratification using subscripts a for age, c for socio-economic group, and g for sex. We used expert judgements to forecast the values of coverage and impact for voluntary reformulation, and of coverage for social marketing and nutrition labelling. In Appendix S2, we present the detailed methods and results of our procedure to elicit these expert forecasts.

Policy	Component	Symbol	Definition
Reformulation	Efficacy	s	Average daily salt intake (g/day).
		b	Proportion of salt intake from processed food.
	Coverage	d	Proportion of products reformulated.
	Impact	η	Proportional reduction of salt content in the products that are reformulated.
Social marketing	Efficacy	s	Experimentally identified effect on salt intake (g/day).
	Coverage	d	Change in proportion exposed to intervention at a level sufficient for persistent behaviour change.
	Impact	η	Proportion exposed who maintain reduced salt.
Nutrition labelling	Efficacy	s	Experimentally identified effect on salt intake (g/day).
	Coverage	d	Change in proportion exposed to label information at point of purchase.
	Impact	η	Proportion exposed at point of purchase who respond by switching or foregoing an item.
	Additional parameters	k	Period in days between shopping events.
		m	Number of processed items bought at each event.
		l	Number of people who share food.

5.1.1 Reformulation of processed foods

We defined processed foods as any product that could potentially be reformulated to reduced salt. The efficacy of reformulation, i.e., its potential effect, depends on the amount of salt in the diet that is obtained from processed foods. We computed this as the average daily salt intake (from the HSfE, Section 1) multiplied by the proportion of salt intake from processed foods (from the LCFS, Section 2). Referring to the symbols in Table 1, this is the product $s \times b$.

The coverage of reformulation describes the proportion of the products consumed by the average individual that experience some extent of reformulation to lower salt content. This extends our formula to $s \times b \times d$.

The impact of reformulation is the average effect on salt content within products that are reformulated. Thus, the final reduction in salt intake (S) due to reformulation is

$$S = s \times b \times d \times \eta. \tag{2}$$

Introducing the stratification used in our model gives,

$$S_{g,a,c} = s_{g,a,c} \times b_a \times d_c \times \eta_c, \tag{3}$$

i.e., policy parameters were stratified by socio-economic group; the proportion of salt from processed foods by age; and salt intake by sex (g), age (a) and socio-economic group (c).

For voluntary reformulation, we obtained data on each parameter in (3), but for mandatory reformulation we reduced (3) to

$$S_{g,a,c} = s_{g,a,c} \times b_a \times \eta, \tag{4}$$

i.e., we fixed the proportion of products reformulated to $d = 1$. We then investigated the effect of fixed levels of η in all socio-economic groups.

5.1.2 Social marketing

For our behaviour change interventions, we first chose a maximum potential effect. The best information on this efficacy for salt intake comes from experimental trials of dietary education and counselling. We used the meta-analysis of Rees et al. [7], who report the long-term effect on sodium intake, which we converted to a salt reduction of 2.39 ± SE 0.47 g/day.

This potential reduction can be realised only by increases in the coverage of each intervention. For social marketing, we defined an individual as exposed to messages if they *receive and understand a message sufficiently that, as a consequence, they might change their behaviour to a persistently reduced salt intake.* Referring to Table 1, we described the reduction in salt intake for a change in coverage by the product, $s \times d$.

Finally, the impact of changes in exposure depends primarily on the degree of individual response, if the stimulus for behaviour change is received. Due to its difficultly of estimation, we investigated the effect of fixing different levels of this parameter in all socio-economic groups. Thus, we modelled the change in salt intake as

$$S = s \times d_c \times \eta. \tag{5}$$

5.1.3 Nutrition labelling

Efficacy, i.e., the maximum potential effect on an individual's salt intake, was the same as for social marketing. We translated this to the potential reduction in the mass of salt in the shopping basket from switching/foregoing one item, assuming that for the full effect, the entire shopping basket is altered from its usual content.

Referring to the symbols in Table 1, we defined a fixed pattern of shopping behaviour by the period in days between shopping events ($k = 7$), the number of processed food items bought at each event ($m = 25$), and the number of people who share the food ($l = 2$).

Using these parameters, the potential reduction in mass of salt (p) in the shopping basket from switching/foregoing one item is

$$p = \frac{s \times k \times l}{m}. \tag{6}$$

We defined changes in the coverage of nutrition labelling in terms of exposure on a per-item basis: an individual is exposed if they *view the label on an item, and understand the label information enough that it could be a useful tool in the self-regulation of daily nutrition.* Thus, exposure covers factors such as label design and customer education.

The impact of this exposure depends on the probability that, if a shopper receives label information, they switch/forego a product that they would otherwise have purchased. As with social marketing, we investigated the effect of different levels of this parameter.

We defined the initial proportion of individuals exposed to information as j, which changes to $j + d$ due to developments in labelling. Then, the effect on an individual's daily salt intake is given (by the mean value of a binomial distribution) as

$$
\begin{aligned}
S &= \frac{p}{kl}\sum_{z=0}^{z=m} z\binom{m}{z}\left[([j+d_c]\eta)^z(1-[j+d_c]\eta)^{(m-z)} - (j\eta)^z(1-j\eta)^{(m-z)}\right], \\
&= \frac{p}{k\times l}\times m\times d_c\times\eta, \\
&= s\times d_c\times\eta. \qquad (7)
\end{aligned}
$$

6 IMPACT$_{\mathbf{SEC}}$: the epidemiology layer

Here, we describe the mathematical structure and data used to complete the linkages of salt intake to SBP, and SBP to CHD death rates. This is the same methodology as previous versions of IMPACT$_{\mathrm{SEC}}$ [8].

6.1 Link 1: Salt intake to SBP

We took this link from the meta-analysis of He et al. [9] (see data in Table 4), who conducted a Cochrane systematic review of randomised trials lasting at least four weeks. The relationship is a linear dose-response that is strongest in hypertensive individuals and weakens with age.

Table 2: **Definitions of symbols used to link the changes in salt intake and SBP**.

Symbol	Definition
S	The change in average daily salt intake (g/day).
H	The change in SBP (in millimeters of mercury, mmHg).
α	The linear dose-response coefficient linking S to H.
z	Hypertension status: z = normotensive or hypertensive.
a	Age.
β	The linear effect of age on the relationship of S to H.
P	Proportion of individuals with hypertension.
c	Socio-economic class : in this study there are five socio-economic groups.
g	sex.

Referring to the symbols in Table 2, the link between changes in salt intake and SBP, for each hypertension-status, is

$$H_{g,a,c,z} = \frac{\alpha_z S_{g,a,c,z} + \beta(a-50)}{6}, \qquad (8)$$

where 50 is the median age and 6 is the change in salt intake (g/day) for which the coefficients are quoted. For each sex, age and socio-economic group, the change in SBP is the weighted average of the changes for each hypertension status, i.e.

$$H = PH_{z=\text{hypertensive}} + (1-P)H_{z=\text{normotensive}}. \qquad (9)$$

He et al. also provide 95% confidences intervals (CIs) around the estimates of α_z and β (Table 4). Assuming that the error around the estimates has a normal distribution, their standard error is

$$\mathrm{SE} = \frac{(\text{upper 95\% CI} - \text{lower 95\% CI})}{2\times 1.96} \qquad (10)$$

and so their error distributions (for uncertainty analysis, Section 7.3) have the form

$$f(x) = \mathcal{N}(x, \mathrm{SE}(x)^2). \qquad (11)$$

6.2 Link 2: SBP to CHD death rates

We took this link from the meta-analysis by the Prospective Studies Collaboration [10], a synthesis of 61 prospective studies containing a million individuals in total (Table 5).

Table 3: **Additional symbols used to link the changes in SBP and CHD death rates**.

Symbol	Definition
M	The original central rate of death from CHD during a particular age interval.
γ	The hazard ratio linking M to a change in SBP of $H = 20$ mm Hg.
θ	The beta-coefficient linking $\ln M$ to a change in SBP of $H = 1$ mm Hg.
M'	The new central rate of death from CHD, following a change in SBP.

We first converted the hazard ratios from a change in SBP of $H = 20$ mm Hg to the change in log CHD death rates for a change in SBP of $H = 1$ mm Hg, using the formula (refer to symbols in Table 3)

$$\theta = -\frac{\ln(\gamma)}{20}. \tag{12}$$

In addition, since the age-intervals in Table 5 did not match the age-intervals we used in our model, we adjusted by fitting linear regressions by sex to the coefficients from (12), and then taking the appropriate fitted values.

With these values, we estimated the new rates of death from CHD due to a change in SBP (for each sex, age and socio-economic), using

$$M'_{g,a,c} = M_{g,a,c} \exp(-H_{g,a,c}\theta_{g,a}). \tag{13}$$

We assumed that the errors around the log of the hazard-ratios have a normal distribution, so their distributions (for uncertainty analysis) are

$$f(\ln \gamma) = \mathcal{N}(\ln \gamma, \mathrm{SE}(\ln \gamma)^2). \tag{14}$$

7 Outputs

Below, we use y to index each year, and N to indicate population numbers.

7.1 Aggregate outcomes and the socio-economic differential

We computed the cumulative number of CHD deaths prevented or postponed over our forecast period using the formula

$$T = -\sum_y \sum_a \sum_g \sum_c N_{y,a,g,c} \times (M'_{y,a,g,c} - M_{y,a,g,c}). \tag{15}$$

For the socio-economic differential in this effect, we first calculated the socio-economic-specific numbers of premature CHD deaths prevented or postponed as

$$T_c = -\sum_y \sum_a \sum_g N_{y,a,g,c} \times (M'_{y,a,g,c} - M_{y,a,g,c}). \tag{16}$$

Next we summarised the distribution of T_c among socio-economic groups using the Slope Index (SI) (Figure 1). The Slope Index fits a linear regression through the socio-economic-specific values, and then reports the difference in deaths prevented or postponed between the least and most deprived.

7.2 Life-years gained

We used data for 2012 from the ONS to compute age, sex and IMDQ specific remaining life expectancies ($e_{a,g,c}$). Remaining life expectancies after age 85 were stratified by sex only. We then computed life-years gained as

$$LYG = \sum_y \sum_a \sum_g \sum_c T_{y,a,g,c} \times e_{a,g,c}. \tag{17}$$

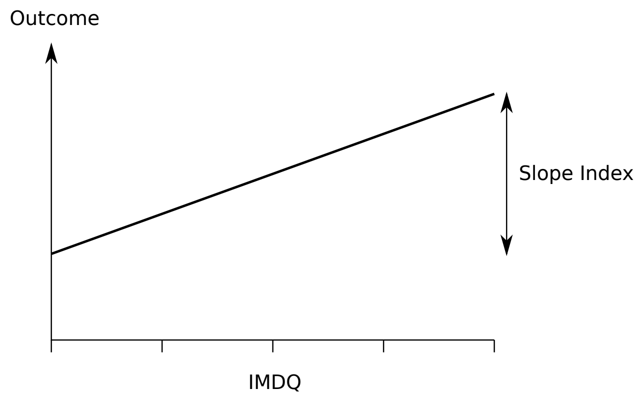

Figure 1: **Illustration of the Slope Index**, as the range of a linear gradient fitted by least-squares regression through the IMDQ-specific values. Positive values indicate a larger effect in the most affluent (IMDQ1); negative values indicate a larger effect in the most deprived (IMDQ5).

7.3 Uncertainty analysis

From our simulated output distributions, we calculated means and 95% prediction intervals. As we detail below (Section 7.3.2), we also used these prediction intervals to investigate the extent to which our experts' judgements contributed to overall uncertainty.

7.3.1 Assumptions of independence in draws of related parameters

When taking Monte Carlo draws from the distributions of the many parameters in our model, attention must be paid to the correlation between draws of related parameters. For example, we fixed a correlation of 1 between draws from different strata of our stratified variables (e.g., salt intake), i.e., we sampled at the population-level rather than from each stratum independently. For our main results, we assumed a correlation of 0 between parameters from the same statistical analysis (e.g., between the average effect and age-adjustment of the salt–SBP relationship, Table 4), and between the best-guess and socio-economic differential of our experts' judgements. For the experts' judgements, since we did not elicit the correlation directly, we tested whether our findings were robust to changing the assumed correlation to 1 instead of 0; although we do not present the results, we note that this had a negligible effect on our findings.

The general method that we used is as follows. Say that r is the correlation between random draws from the distributions of different parameters. Then define n to be the number of different parameters. So, if we compute

$$B = \sqrt{r} \times x, \tag{18}$$

where x is drawn from a standard normal distribution, i.e.,

$$x \sim \mathcal{N}(0, 1), \tag{19}$$

and then compute

$$\mathbf{y} = B + \sqrt{(1 - r)} \times \mathbf{v}, \tag{20}$$

where $\mathbf{v}$ is vector of length n with each element drawn from a standard normal distribution, we get

$$\mathbf{z} = \frac{1}{\sqrt{2\pi}} \exp\left(-\frac{\mathbf{y}^2}{2}\right), \tag{21}$$

which is a vector of length n that gives the point in cumulative distribution of each parameter at which we should take our sample.

7.3.2 Uncertainty decomposition

Finally, for each policy option, we investigated the extent to which our experts' judgements contributed to the overall uncertainty in our outcomes. To do so, we quantified uncertainty by the width of the 95% prediction intervals of the total number and socio-economic differential of CHD deaths prevented or postponed. We first computed these intervals with all sources of uncertainty, then removed the declared uncertainty around our experts' judgements by re-estimating our model with the mean expert judgements. We then calculated their percentage contribution to total uncertainty [11].

8 Data tables

Table 4: **Values taken from He et al. [9]**. α is the effect on SBP of a 6 g/day change in salt intake. β is the consequence for this effect of increasing age.

Parameter	Estimate	95% CI
$\alpha_{z=\text{hypertensive}}$	5.39	4.15, 6.62
$\alpha_{z=\text{normotensive}}$	2.42	1.29, 3.56
β	−0.06	−0.116, −0.006

Table 5: **Values taken from the Prospective Studies Collaboration [10]**. The relationship varies by both sex and age; effects are quoted as hazard ratios that give the change in death rates for an SBP reduction of 20 mmHg

sex	Age	Hazard ratio	95% CI
Female	40-49	0.40	0.32, 0.49
	50-59	0.49	0.45, 0.54
	60-69	0.50	0.47, 0.53
	70-79	0.55	0.53, 0.58
	80-89	0.64	0.60, 0.68
Male	40-49	0.50	0.46, 0.54
	50-59	0.50	0.49, 0.52
	60-69	0.55	0.54, 0.57
	70-79	0.62	0.60, 0.64
	80-89	0.69	0.65, 0.73

References

[1] Craig R, Mindell J. Health Survey for England; 2011.

[2] Sadler K, Nicholson S, Steer T, Gill V, Bates B, Tipping S, et al. National diet and nutrition survey - assessment of dietary sodium in adults (aged 19 to 64 years) in England, 2011; 2012.

[3] Bajekal M, Scholes S, O'Flaherty M, Raine R, Norman P, Capewell S. Unequal trends in coronary heart disease mortality by socioeconomic circumstances, England 1982–2006: an analytical study. Plos One. 2013;8(3).

[4] Allen K, Gillespie DOS, Guzman Castillo M, Diggle PJ, Capewell S, O'Flaherty M. Premature coronary heart disease deaths in England: predicting future trends and inequalities. unpublished manuscript;.

[5] Schmid VJ, Held L. Bayesian age-period-cohort modeling and prediction - BAMP. Journal of Statistical Software. 2007;21(8):1–15.

[6] Tugwell P, de Savigny D, Hawker G, Robinson V. Applying clinical epidemiological methods to health equity: the equity effectiveness loop. British Medical Journal. 2006;332(7537):358–361.

[7] Rees K, Dyakova M, Wilson N, Ward K, Thorogood M, Brunner E. Dietary advice for reducing cardiovascular risk. The Cochrane database of systematic reviews. 2013;12:CD002128–CD002128.

[8] Bajekal M, Scholes S, Love H, Hawkins N, O'Flaherty M, Raine R, et al. Analysing recent socio-economic trends in coronary heart disease mortality in England, 2000-2007: a population modelling study. PLOS Medicine. 2012;9(6):e1001237.

[9] He FJ, Li JF, MacGregor GA. Effect of longer term modest salt reduction on blood pressure: Cochrane systematic review and meta-analysis of randomised trials. British Medical Journal. 2013;346:f1325.

[10] Prospective Studies Collaboration. Age-specific relevance of usual blood pressure to vascular mortality: a meta-analysis of individual data for one million adults in 61 prospective studies. Lancet. 2002;360(9349):1903–1913.

[11] O'Hagan A. Probabilistic uncertainty specification: Overview, elaboration techniques and their application to a mechanistic model of carbon flux. Environmental Modelling & Software. 2012;36:35–48.
