# Supplementary material for: The Health Equity and Effectiveness of Policy Options to Reduce Dietary Salt Intake in England: Policy Forecast
Source: PLoS One. 2015 Jul 1;10(7):e0127927. doi: 10.1371/journal.pone.0127927 (PMC4488881; doi:10.1371/journal.pone.0127927)
Supplement: S2 Appendix — (PDF) [file pone.0127927.s002.pdf]

Appendix 2 for "The health equity and effectiveness of policy options to reduce dietary salt intake in England: policy forecast"

Duncan Gillespie, Kirk Allen, Maria Guzman-Castillo, Piotr Bandosz, Patricia Moreira, Rory McGill, Elspeth Anwar, Ffion Lloyd-Williams, Helen Bromley, Peter Diggle, Simon Capewell, and Martin O'Flaherty

ELICITATION OF EXPERT FORECASTS THROUGH A QUESTIONNAIRE

In this appendix, we provide further details on the expert elicitation part of our modelling method, through which we derive a forecast of potential policy outcomes for dietary salt intake. As described in the main manuscript, we frame our approach around our model of the steps by which different policies arrive at their effect on dietary salt intake. The step that we have made the focus of our expert elicitation is the future implementation of policy options. By "implementation" we mean any practical aspects such as the proportion of products that are reformulated to reduce salt, or the proportion of the population reached by an information campaign. We have described our sample of experts in the main manuscript. Here, we address two main issues.

First, we developed a questionnaire with which to elicit the experts' opinions about future implementation, and about the potential socio-economic differentials in future implementation. In many contexts, it might be better to conduct interviews with experts than to send them a questionnaire to interpret by themselves, with dangers of misunderstanding. For this reason, it is common practice that expert elicitation asks for substantial time investments from the participants. Nevertheless, having known in advance who our experts would be, and that they are time-constrained, we believed that the most sensible approach was to construct a short (< 10 minutes) questionnaire to be completed in a free moment. We pre-piloted our questionnaire to shorten and refine our questions so that they might be quickly understandable. The result was a one-page questionnaire with 7 questions that asked for each expert's min, max and best guess (S3 Appendix).

Second, we employed a suitable way to combine opinions elicited in this way from several experts to be used as the basis for our epidemiological forecasts (detailed in S1 Appendix). Pooling expert opinions means to merge many individual's probability distributions on unknown values into a single collective probability distribution. The natural way to convert min, max and best-guess data into a probability distribution is to input these as parameters to the Program Evaluation and Review Technique (PERT) distribution [1]. Since we had no basis on which to weight each expert's judgement, we proceeded by unweighted classical linear pooling. In our procedure, we first sampled an expert at random and second drew a sample from their personal probability distribution for each value.

The scenarios

In Table 1 we describe our model of the steps by which different types of policy might produce an effect on dietary salt intake. In Table 2 we present the questions that

we constructed around the parameters that concerned policy implementation to elicit forecasts from our experts.

We prefaced our questionnaire with the following brief introduction:

As you know, the FSA[1] Salt Reduction Programme was followed by a decrease in salt intake of about 1.5g/day in adults. There has been subsequent discussion about continuing this strategy, about additional or alternative approaches, and also about potential effects on inequalities.
So where next?? The start of the new UK government in 2015 will be an important moment to re-assess policies to reduce salt intake.

Our questions asked the experts to consider their expectations of a future salt reduction programme, starting from 2015. In this context, the major (non-fiscal) policy options are:

- **Product reformulation**: Engagement with industry to promote voluntary reductions of salt-content under the Department of Health's Responsibility Deal [2]. The scenario we presented did not specify any particular food group(s).
- **Label information**: Increasing the presence, visibility, and utility of nutrition information [3]. The experts were free to consider information presented in any particular form(s), e.g., front-of-pack traffic light labelling or daily intake guidelines.
- **Social marketing**: The population-wide provision of information / education on healthy diet and lifestyle. In our scenario, the experts were free to consider implementation via mass media, leaflets, outreach activities, or all of these as in the UK's Change4Life information campaign [4].

We asked the experts to consider the future policy implementation from the perspective of the English adult population. We specified a relatively short forecast time horizon of 2020, so as to make changes by the time horizon easier to imagine. Note that we ran our epidemiological forecast up to 2025, holding the level of implementation constant from 2020.

For each question, we asked the experts to provide "your best estimate, and also to suggest the absolute minimum value and maximum which you think could be true, include all possibilities except those that you consider extremely unlikely (less than 1% chance)". We first asked the experts to provide a forecast value for the population as a whole. We then asked for their expectation of the socio-economic differential in their forecast values, with socio-economic variation defined by quintiles of the Index of Multiple Deprivation[2]. We elicited this differential as a value relative to 1, with the question:

[1] UK Food Standards Agency.
[2] The Index of Multiple Deprivation (IMD) summarises the characteristics of geographical (lower layer super output) areas based on income, employment, health, education, crime, access to services and living environment.

If the effect is represented by 1.00 in the least deprived (richest) social group, what would be the value in the most deprived (poorest) group? (Putting "1.0" would mean no different, "0.2" would mean just 20% of that in the richest.)

We also gave the example:

If I think the value might perhaps be about 50% lower in the poorest fifth, compared with the richest I would make my BEST estimate 0.5. Furthermore, I would acknowledge a degree of personal uncertainty, and suggest the value might be as low as 0.2 (minimum), or as high as 0.9 (maximum). In other words, I think the true value in the poorest is definitely somewhere between 20% and 90% of the value in the richest.

Table 1. Our model of the steps to policy outcome. For each policy option we outline the steps and data sources, highlighting the steps that were forecast by our topic experts. The steps in the model: efficacy, coverage and impact are the parameters s, d and η that are defined extensively in section 5.1 of S1 Appendix.

**Policy option**	**Step in policy model**	**Data source**
Voluntary reformulation	Efficacy: the initial dietary salt intake from processed foods.	Survey data on the average daily intake of dietary salt and proportion of this intake that derived from processed foods.
	Coverage: the percentage of products included in future reformulation.	**Forecast by experts**.
	Impact: the percentage reduction in salt content among these products	**Forecast by experts**.
Nutrition labelling & social marketing	Efficacy: maximum potential effect.	From published meta-analysis of the long-term effect of dietary counselling.
	Coverage: the change in the percentage of individuals exposed to information, who understand it sufficiently that persistent behaviour change could occur.	**Forecast by experts.**<br>We asked experts to assume 10% initial coverage across all social groups.
	Impact: the proportion of the maximum potential effect (efficacy) that is realised.<br>This parameter depends on variation in individual responsiveness.	Due to lack of information, we computed our forecast at a range of values.

Table 2. The 7 questions from our questionnaire (S3 Appendix). Note that for labelling and social marketing, we first anchored experts by asking them to assume, arbitrarily, that coverage was currently 10% in all deprivation quintiles. We then asked them to estimate the future coverage in 2020.

Policy option	Additional information provided on the questionnaire	Question text
Reformulation to reduce salt content.	Reducing the amount of salt added by industry to processed foods (including in the catering sector).	(A: Coverage) What percentage of the processed food products currently consumed by an average English adult are likely to be reformulated to reduce salt by 2020?
		(B: Impact) In the processed foods which ARE reformulated to reduce salt by 2020, what percentage reduction is likely to be achieved?
		(C: Inequality) If the combined value of your estimates in (A) and (B) was represented by 1.00 in the richest, what do you think would the value be in the poorest? (Putting "1.0" would mean no different, "0.2" would mean just 20% of that in the richest)
Social marketing	Healthy eating education and promotion, ranging from low intensity messages like the generic "Change4Life" campaign to the salt specific "Sid the Slug" and "Cut the Salt" campaigns.<br>We define an adult as "EXPOSED" if they RECEIVE and UNDERSTAND a message sufficiently that they persistently REDUCE their salt intake.	(A: Coverage) Assuming that [10]% of the entire population of adults are currently sufficiently EXPOSED to messages for PERSISTENT behaviour change, what is this percentage likely to be in 2020?
		(B: Inequality) If the change in EXPOSURE in (A) is represented by 1.00 in the richest, what might be the value in the poorest?
Labelling of nutritional content	Any nutrition information available at the decision to purchase a processed food product.<br>We define a customer as "EXPOSED" to nutrition labelling if they VIEW the label on an item, and UNDERSTAND the information sufficiently to CHANGE their purchasing behaviour to REDUCE their salt intake.	(A: Coverage) Assuming that [10]% of the entire population of adults are currently EXPOSED to label information when they consider purchasing a processed food product, what is this percentage likely to be in 2020?
		(B: Inequality) If the change in exposure in (A) is represented by 1.00 in the richest, what would the value be in the poorest?

Elaborating the experts' judgements to probability distributions

We conducted all analyses in the R environment (version 3.1.0) [5]. For each expert, we converted each set of best, min and max estimates into a probability distribution over the potential values using a standard PERT distribution (Figure 1). We then took 10,000 samples from these distributions, first sampling an expert at random and then sampling probabilistically from their probability distributions.

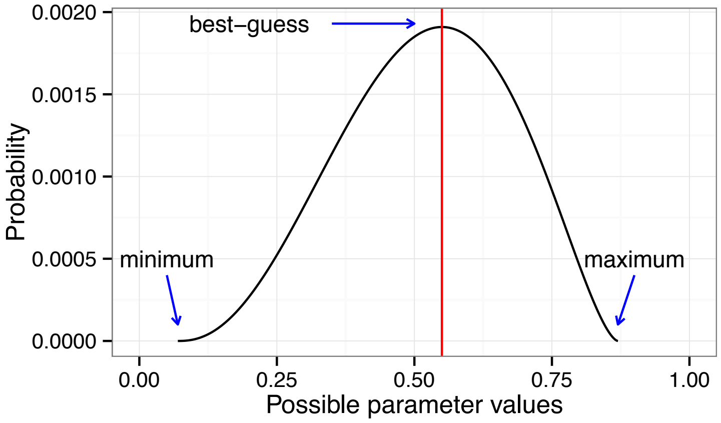

Figure 1. Probability distribution generated from expert judgements. We used the PERT distribution (a special form of a beta distribution) to produce a smooth distribution peaked on the best guess (vertical red line), ranging from the minimum to maximum values.

In our questionnaire the socio-economic differential specified by our experts followed a linear gradient across socio-economic groups, which we described with a simple control parameter (a value in the poorest relative to 1 in the richest).

We took each expert's forecast for the population as a whole to be the value for IMDQ3. We then defined a gradient between IMDQ1 and IMDQ5 in such a way that it passed through the effect in IMDQ3. The value for IMDQ2 is therefore the mean of IMDQ1 and IMDQ3, and IMDQ4 is the mean of IMDQ3 and IMDQ5.

Say our socio-economic groups are indexed $c = 1, ..., n$, and the control parameter β gives the socio-economic differential in a variable x of length n. If x_1 is the value in the least deprived, and x_n is the value in the most deprived, our control parameter is defined by

$$x_n = x_1[1+\beta],$$

i.e., as the proportional difference between the least and most deprived groups. This means that the control parameter is related to the value (x^*) in the median socio-economic group by

$$x^* = x_1[2+\beta]/2,$$

so

$x_1 = 2x^*/(2+\beta)$,

and

$x_n = 2x^*(1+\beta)/(2+\beta)$.

Based on these formulae, we elaborated the experts' estimates into socio-economic-specific values for use in our model. Note that if x has limits between 0 and 1, there are certain limits on the values that x^* and β can take simultaneously such that x_1 and x_n are also between 0 and 1.

DETAILED RESULTS

Gathering the responses

Of the 25 experts approached, 17 initially agreed to participate. After viewing the questionnaire, a further 4 declined to participate, giving us 13 complete responses (12 by email, 1 over the phone). The reasons for declining to participate after having seen the questionnaire centred on not feeling able to provide a useful future prediction. None of the experts declared a conflict of interest. Only two experts annotated their questionnaires. We asked for further explanation from one expert following an outlying prediction.

The information gathered

As can be seen from Figures 2 and 3, the overall message was one of high prediction uncertainty. For reformulation, the experts predicted that by 2020, an average 39% of products would be reformulated to reduced-salt (Figure 2a). However, there was an extremely wide range of opinion, as illustrated by the 95% prediction interval that ranged from 9% to 82%. In the products reformulated, the experts expected that salt content would fall on average by 24%, but with a prediction interval that ranged from 9% to 46% (Figure 2b).

In terms of the socio-economic differential of effect, the experts predicted that if progress in the most affluent were 1.0, then progress in the most deprived would be on average 0.8, i.e., 20% less (Figure 2c). However, opinions were split on both magnitude and direction of this differential, with the prediction interval ranging from 80% less progress for the most deprived, to 50% more progress.

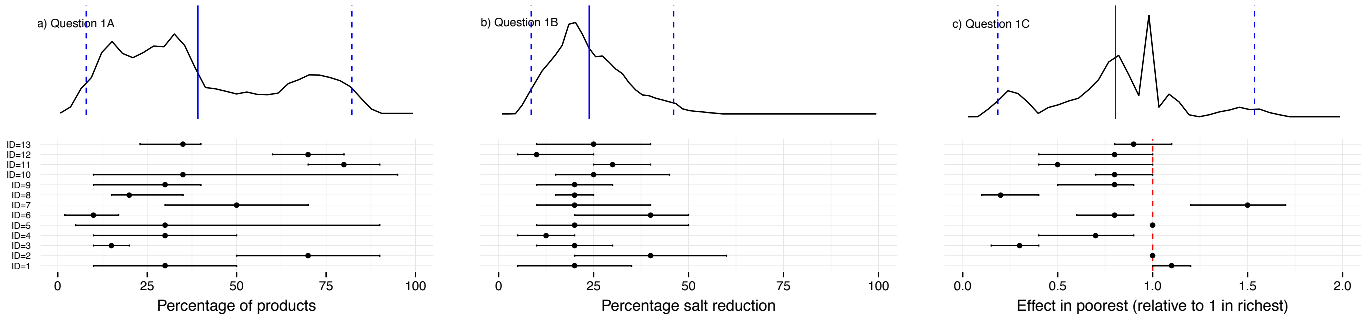

Figure 2. Voluntary reformulation: expected future progress. The individual expert judgements (bottom panels), summarised into pooled probability distributions (top panels). In the top panels, the solid vertical line indicates the pooled mean value, and the broken vertical lines indicate the pooled lower and upper 95% prediction intervals. Expert judgements were given as best-guess, minimum and maximum values for: (a) the percentage of processed foods likely to be reformulated by 2020; (b) the percentage salt reduction in these products; (c) the socio-economic differential in (a) and (b) combined. This differential was elicited in the form of a linear gradient across social groups, characterised by the effect in the most deprived relative to 1.0 in the most affluent.

For nutrition labelling and social marketing, bearing in mind that we first asked the experts to assume a starting point of 10% initial population coverage, the average prediction was that by 2020, coverage would reach 23% for labelling, and 21% for social marketing (Figure 3a, b). Again there was a wide split of opinion on the magnitude and direction of this change, as shown by the prediction intervals of 5% to 50% (compared to the 10% baseline).

The predicted socio-economic differential of the change in coverage was also similar for each intervention, indicating a common thought process: if progress in the most affluent were represented by 1.0, then progress in the most deprived would be on average 0.5, i.e., 50% less (Figure 3c, d). For nutrition labelling, the prediction interval on the socio-economic differential also included the potential of more change among the most deprived.

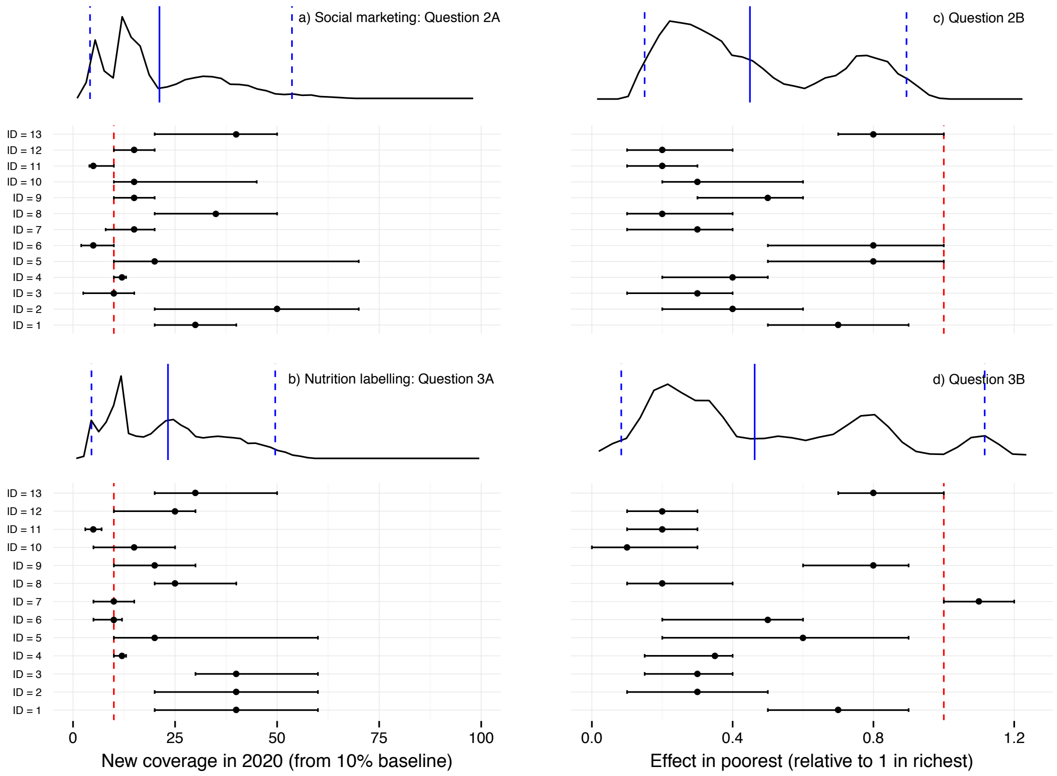

Figure 3. Social marketing and nutrition labelling: expected future progress. The expert judgements for changes in the coverage of social marketing (top panels) and nutrition labelling (bottom panels) by 2020. In the top panels, the solid vertical line indicates the pooled mean value, and the broken vertical lines indicate the pooled lower and upper 95% prediction intervals. In the bottom panels, the broken vertical lines indicate the baseline for the expert's subsequent judgement. Expert judgements were given as best-guess, minimum and maximum values for: (a & b) the new percentage coverage in 2020, from a supposed baseline of 10% in all social groups; (c & d) the socio-economic differential in this change, characterised by the value in the most deprived if the value in the most affluent is 1.0.

The estimates elaborated by socio-economic group
The results of pooling the experts' answers, and elaborating them to values specific to each Index of Multiple Deprivation quintile indicated clearly that our experts expected that future implementation would reach more deprived groups less well (Figure 4).

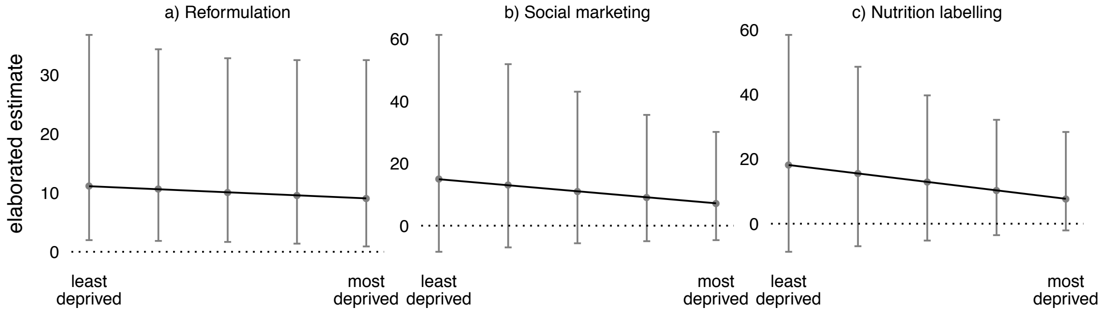

Figure 4. The experts' forecasts of policy implementation elaborated to values specific to each Index of Multiple Deprivation quintile. For a) Reformulation to reduced salt, values are the combination of the estimated percentage of products that might see some reformulation by 2020, the consequent percentage reduction in salt content, and the estimated socio-economic differential in these two estimates combined. The values are therefore the expected percentage reduction in the salt content of products consumed by each socio-economic group. For b) social marketing and c) nutrition labelling values are the forecast change by 2020 in the percentage of individuals who are exposed to information, according to the definition of exposure for each policy option given in Table 2. Error bars show the 95% prediction intervals around the mean estimate.

REFERENCES

1. Clark CE: **The PERT model for the distribution of an activity.** *Operations Research* 1962, **10**:405–406.
2. Jebb SA: **The Public Health Responsibility Deal Food Network**. *Nutrition Bulletin* 2012, **37**(4):355–358.
3. Hawley KL, Roberto CA, Bragg MA, Liu PJ, Schwartz MB, Brownell KD: **The science on front-of-package food labels**. *Public Health Nutrition* 2013, **16**(3):430-439.
4. Department of Health: **Change4Life Marketing Strategy**. 2009.
5. R Core Team: **R: A language and environment for statistical computing.** Vienna, Austria: R Foundation for Statistical Computing; 2014.
