# Supplementary material for: The Health Equity and Effectiveness of Policy Options to Reduce Dietary Salt Intake in England: Policy Forecast
Source: PLoS One. 2015 Jul 1;10(7):e0127927. doi: 10.1371/journal.pone.0127927 (PMC4488881; doi:10.1371/journal.pone.0127927)
Supplement: S3 Appendix — (PDF) [file pone.0127927.s003.pdf]

Study on the effectiveness of interventions to reduce salt intake, and potential inequalities

Greetings!

As you know, the FSA Salt Reduction Programme was followed by a decrease in salt intake of about 1.5g/day in adults. There has been subsequent discussion about continuing this strategy, about additional or alternative approaches, and also about potential effects on inequalities.

So where next?? The start of the new UK government in 2015 will be an important moment to re-assess policies to reduce salt intake.

We would like to ask you, as a person with recognized expertise in food policy, to help us by answering the simple questions in this brief (1 page) questionnaire. We will use your responses to build a simple epidemiological model. (We would be happy to feed back the results if you wish).

The major (non-fiscal) policy options include <u>Reformulation</u>, <u>Label information</u> & <u>Social marketing</u>

The 7 questions seek your opinion on each intervention, the likely reach, effectiveness and potential consequences for inequalities (differences by Index of Multiple Deprivation quintiles)

The 7 questions ask you to provide your best estimate, and also to suggest the absolute minimum value and maximum which you think could be true, <u>include all possibilities except those that you consider extremely unlikely</u> (less than 1% chance).

Please feel free to offer comments or your reasons for your estimates to each question

Estimates are asked for either as percentages or (for the inequality estimates) as values relative to 1.00. **For example:**

> *If the effect is represented by **1.00** in the least deprived social group, what would be the value in the most deprived group?*
>
> If I think the value might perhaps be about 50% lower in the poorest fifth, compared with the richest I would make my BEST estimate **0.5.** Furthermore, I would acknowledge a degree of personal uncertainty, and suggest the value might be as low as **0.2** (minimum), or as high as **0.9** (maximum). In other words, I think the true value in the poorest is definitely somewhere between 20% and 90% of the value in the richest.

Thank you very much for helping us

Very Best Wishes

Duncan Gillespie, Martin O'Flaherty and Simon Capewell

Questionnaire The effectiveness of interventions to reduce salt intake

1. Reformulation to reduce salt content

Reducing the amount of salt added by industry to processed foods (including in the catering sector).

(A) What percentage of the processed food products currently consumed by an average English adult are likely to be reformulated to reduce salt by 2020?

BEST ESTIMATE (%):	Minimum (%):	Maximum (%):

(B) In the processed foods which ARE reformulated to reduce salt by 2020, what percentage reduction is likely to be achieved?

BEST ESTIMATE (%):	Minimum (%):	Maximum (%):

(C) If the combined value of your estimates in (A) and (B) was represented by 1.00 in the richest, what do you think would the value be in the poorest? *(Putting "1.0" would mean no different, "0.2" would mean just 20% of that in the richest)*

BEST ESTIMATE (relative to 1):	Minimum value (relative to 1):	Maximum value (relative to 1):

2. Social marketing (Healthy eating education and promotion, *ranging from Low intensity messages like the generic "Change4Life" campaign to the salt specific "Sid the Slug" and "Cut the Salt" campaigns).*

- We define an adult as "EXPOSED" if they RECEIVE and UNDERSTAND a message sufficiently that they persistently REDUCE their salt intake.

(A) Assuming that [10]% of the entire population of adults are currently sufficiently EXPOSED to messages for PERSISTENT behaviour change, what is this percentage likely to be in 2020?

BEST ESTIMATE (%):	Minimum (%):	Maximum (%):

(B) **If the change in EXPOSURE in (A) is represented by 1.00 in the richest, what might be the value in the poorest?**

BEST ESTIMATE (relative to 1):	Minimum value (relative to 1):	Maximum value (relative to 1):

3. Labelling of nutritional content *(Any nutrition information available at the decision to purchase a processed food product).*

We define a customer as "EXPOSED" to nutrition labelling if they VIEW the label on an item, and UNDERSTAND the information sufficiently to CHANGE their purchasing behaviour to REDUCE their salt intake.

(A) Assuming that [10]% of the entire population of adults are currently EXPOSED to label information when they consider purchasing a processed food product, what is this percentage likely to be in 2020?

BEST ESTIMATE (%):	Minimum (%):	Maximum (%):

(B) If the change in exposure in (A) is represented by 1.00 in the richest, what would the value be in the poorest?

BEST ESTIMATE (relative to 1):	Minimum value (relative to 1):	Maximum value (relative to 1):

THANK YOU VERY MUCH FOR COMPLETING THIS SURVEY!!

Overleaf, we ask that you complete the permission, confidentiality and competing interests form.

Permission and Confidentiality

We are very grateful for your participation in this exercise. Please sign this form to confirm your agreement.

1. In participating, you acknowledge that you are an expert in the field of policy on the reduction of salt intake and were invited to this process through peer-nomination.
2. You give us permission to use your estimates in our modelling exercise, which will lead to publication in a peer-reviewed journal.
3. You acknowledge that you will not be linked personally with any specific estimate and that your association with this exercise will be anonymous. However, we would be happy to acknowledge your contribution on publications that include your estimates, if you so wish.
4. We assume that you have no competing interests, i.e., financial links with the food or salt industries. However, please do tell us if you feel that there might be any competing interests.

I am happy to support this research project on the conditions detailed above

……………………………………………..

(Signed)

……………………………………………..

Date
